# Lectures on the Theory and Practice of Physic

**Published:** 1845-02

**Authors:** 


### REVIEWS.

ART. II.—*Lectures on the Theory and Practice of Physic.*— By JOHN BELL, M.D., Fellow of the College of Physicians Philadelphia; Corresponding Secretary of the Philadelphia Medical College; Member of the American Philosophical Society, and of the Georgofili Society of Florence, &c., &c., and by WILLIAM STOKES, M.D., Lecturer at the Medical School, Park street, Dublin; Physician to the Meath Hospital, &c., &c. *Third Edition, enlarged and improved.* 2 vols. 12mo. Philadelphia: Ed. Barrington & Geo. D. Haswell. 1845. pp. 728 and 790.

The alterations and improvements in this edition of these Lectures, consist in the omission of the Treatise of Dr. Stokes on the *Diagnosis and Treatment of Diseases of the Chest*, and the introduction of matter relating to the diseases of the organs of generation in both sexes, to several diseases of the respiratory apparatus, omitted before, to tubercular meningitis, epidemic meningitis, scrofula, syphilis, the exanthemata, rheumatism and gout, amounting, altogether, to about three hundred pages. Of the large accumulations made of late years on the pathology and modified treatment of the Diseases of Children, Dr. Bell has made profitable use, and this is a portion of this valuable work which will attract the particular attention of the practitioner. We propose to give some account of the matters embraced under that head in this article, and we commence with a quotation from the lecture, by Dr. Bell, on the Diseases of Dentition. It is hygienic in its character, and although it conveys no new precept, the lesson it impresses is one of great consideration, as thus laid down:

"The plain hygienic precept for avoiding many of the evils which I have just sketched, is, to protect the child by suitable clothing, and with this view to cover all parts of the skin which in after life are kept covered. The breast and shoulders and arms ought to be clothed, as they are among the most susceptible parts of the body to atmospherical vicissitudes and extremes. The common and hackneyed but ill-founded excuse of a wish to harden the child, is not applicable to the practice of leaving naked and exposed these portions of the body; for, it is not meant that they should remain so after the child leaves the nursery, nor ever be so subsequently in any period of after life; at any rate in the male sex. In the texture of the garments we shall be guided by the season; as to fashion, they ought to be always loose.

It may perhaps be said that these considerations and details belong to the nursery, and are beneath the consideration of a professor of the theory and practice of physic. But a little reflexion will soon convince you, without any argument from me, that the theory of medicine involves, in fact imperatively requires, a study of all the probable causes of disease, and of the circumstances which give them additional activity, or in any way modify them. As the highest and noblest aim of ethical philosophy is premonition and prevention, so in medical philosophy it is both more humane and more intellectual to devise means to guard against disease, than to display skill, learning, and research, in trials to cure, uncertain as we must be of a successful result, and knowing often that our best devised efforts, in some cases, will almost certainly fail."

One of the most troublesome of the complaints of young infants is colic, the treatment of which is thus given by Dr. Bell:

"*Infantile colic* is sometimes of the stercoraceous variety; but more frequently it depends on morbid secretions from the liver and bowels, and on imperfect change which the food undergoes from its want of adaptation to the digestive sensibility. Of the first kind is the colic of new-born infants, or when the viscid meconium adheres to the colon, and is not evacuated. Castor oil warmed, and in doses of half a drachm to a drachm, and in more obstinate cases of retention with the addition of five drops of oil of turpentine, will generally suffice to give relief in a case of this nature. Dr. Dewees (*On the Physical and Medical Treatment of Children,*) details a case of the disease, which, by the way, was not colic, caused by retention of the meconium, and in which the common laxatives, castor oil and magnesia, failed to operate. He succeeded at last by the administration of a grain of the carbonate of soda, dissolved in a teaspoonful of lukewarm water, every fifteen minutes, until ten grains were taken. Another modification of stercoraceous colic is met with in infants who are habitually constipated, and whose appetite and growth are both vigorous. Purgatives, as is soon discovered, are not the remedy in this case. We must be content to palliate until, with time and some natural change in the functions, the bad habit is changed. Laxative enemata of the simple kind, or occasionally a little castor oil, or spiced syrup of rhubarb, or manna dissolved in its food, as sweatening, if the child uses spoon-victuals, will generally suffice. A suppository of soap is occasionally useful. I have sometimes given from a quarter to half a grain or a grain of calomel with a little magnesia; but of course not frequently, still less habitually, in every case. This prescription is called for when colic proceeds from deficient secretion of bile, as in jaundice. In this disease, as it attacks new-born infants, I have had occasion to be much pleased with the oil of turpentine, in doses of from ten to twenty drops, with a teaspoonful of castor or sweet oil, repeated at an interval of twenty-four or forty-eight hours. It is, also, one of the best medicines for infantile colic with constipation. Simple syrup, with a little of some essential oil, answers well at times, as follows:—Simple syrup, one pint; oil of rue, eight to ten drops. Mix. Dose, one to two teaspoonfuls.

"In the other, and still more common, colic of children, depending on indigestion, our attention must be first directed to the health of the mother. In her, bad digestion or other derangement of health, kept up sometimes by gross and improper food, drinking tea and coffee to excess and malt liquors; sometimes want of air and exercise, and late hours; and again, by indulgence in strong emotions, or by any cause which irritates the nervous system, must we seek an explanation of the depraved nature of her milk, and, consequently, of colic and other forms of indigestion of the infant. Doctor Dewees (*op. cit.*) relates a case of serious and alarming disease of a child, beginning with colic and running on to vomiting and diarrhœa with great emaciation, which was produced by the altered quality of its mother's milk, owing to severe and protracted toothache. To the child itself suffering from colic, a few grains of carbonate of magnesia, with some simple carminative,—mint, or peppermint water, or camphor mixture, or a grain of subcarbonate of potassa, or two or three drops of liquor potassæ in a similar fluid, with sugar,—will often give relief, without interfering with the peristaltic actions of the bowels, or impairing the digestive energy, as all cordials and mixtures into which opium enters are so apt to do. *Calamus aromaticus*, in powder, with chalk or magnesia, answers a similar intention. In some extreme cases of suffering, a drop or two of laudanum will give the desired relief; but never ought the physician to prescribe it regularly, or to allow of its regular use in the nursery. He can hardly be too emphatic in his cautions against the dangers of the practice of habitual laudanum or opium-taking in child or adult.

"In some cases, infantile colic recurs so regularly at particular times in twenty-four hours as to force attention to periodicity, and to suggest its being treated accordingly. I have, in cases of this nature, given the sulphate of quinia in a dose of twelfth to an eighth of a grain in solution, with the effect of greatly mitigating the violence of the attack, and sometimes of warding it off entirely. One may, however, reasonably suspect, that this periodical colic is sometimes owing to the recurrence of an external exciting cause, as in the quality of the mother's milk at a particular time in the twenty-four hours, rather than to an internal organic condition of the nervous system which generally gives rise to periodicity. In confirmation of this, I may state one of my boarding-school reminiscences. A boy, whose digestive organs were never very strong, used to have regularly every Monday afternoon an attack of colic; not very severe, it is true, but quite troublesome and well-marked in all its symptoms. The cause of this weekly return of disease was almost forced on his attention, after a while, by its uniformly following a dinner on cold beef, which was the regular dish of meat for Monday. Many a dyspeptic, who thinks that his sufferings are entailed on him, of necessity, for life would discover, by a little retrospection of his diet during the preceding twenty-four hours, that these are avoidable; and that if he were to omit some article of the *cold-beef* class, he would escape his special ailment.

5

"Before dismissing the treatment of infantile colic, let me enjoin attention to the feet of the child being carefully covered with warm socks and shoes, which ought to occasionally in the day to be taken off, and the feet rubbed by the warm hands of its mother or nurse before the fire, or over a flue of hot air. The early use of a tepid salt-water bath, to be followed by careful friction over the abdomen and the lower limbs, will prove to be a useful preventive of colic, as well as corroborant of the system generally."

Dr. Bell devotes three lectures to the consideration of that formidable malady of the young, *cholera infantum.* Under the head of *causes* he gives the following interesting details:

"*Causes.*—Physicians are generally agreed as to the obvious causes of cholera infantum. Some are inclined to add not only a less appreciable but at all times a very doubtful and in the present case, a very improbable cause—malaria. The disease appears in our cities with the first heat of summer, and continues through the months of July and August into September; but abating in this latter month, as the weather becomes cooler. But although thus manifestly connected with high atmospherical heat, cholera infantum cannot be said to be the product of this alone; for otherwise it ought to increase in violence in proportion to the warmth of the climate, or as we advance south; which is not the case. Such ought to be the state of things, also, if malaria were the cause. We believe it will be found that it is a more common disease in Boston, for example, than in Charleston; certainly it is more so in Philadelphia than in New Orleans. The explanation I take to be this: that the system of a child, on the approach of its first and even second summer, is very much in the same condition as that of a person newly arrived from the north at a southern city. The susceptibility to heat being great, its effects are felt more sensibly on the nervous system, which it excites, and through it on the vascular system, and even still more, the tegumentary; the skin first and afterwards the mucous membranes. These last are kept in a state of irritation short of phlogosis by the high and continued heat acting on the skin; and the digestive ones in their turn, transmit the irritation to the liver, which is often excited in consequence. Now, if in this state of predisposition the person be exposed to close and impure air, the circulation and nervous system become more and more disturbed, and there is the very imminence of violent disease, which only requires for its coming on an excess or irregularity in food, loss of rest, or any morbid excitement of the nervous system, whether from bodily pain or mental anxiety. In a child, the disease will be cholera; in an adult, bilious remittent fever, or yellow fever. In both there will be great gastro-enteric disorder, with hepatic and cerebral complications.

"In recurring to the original proposition, that the disease is brought on by high atmospherical heat in our cities, we cannot overlook the fact of this being the chief and main endemial agent,—without which other coinciding causes, such as the irritation of teething and of indigestible food, would be generally insufficient for its production. It is, however, the high heat following winter's cold, acting for the first time on an infant whose functions have barely acquired the necessary rhythm, certainly are not accustomed to such stimulation. An infant, exposed from birth to a mild temperature, might be expected to feel less even the great heat of our cities during the summer months. Atmospherical agency is made manifest in the amelioration of existing cases of the disease, and fewer fresh attacks during the interval of a few cool days at any time in the summer; and, on the other hand, augmentation in both during great heats, and particularly during a close and damp state of the air, when the thermometer is at the same time high.

"In a table now before me, I find the number of deaths from cholera infantum in Philadelphia, during a period of ten years (from 1823 to 1831), to be 2323, or on an average 232; the maximum having been in 1831, or 303, and the minimum in 1824, or 155; and those from cholera morbus during the same period, of subjects over ten years of age, 114. You will have observed, in my lecture on epidemic cholera, that the cases of infantile cholera were augmenred in 1832, the 'cholera year,' to 316 in 3 months, as were, indeed, all the diseases of the bowels in that year. In 1833 the amount was one hundred and ninety-seven, or less than it had been for eight years preceding.

"The influence of a tropical climate, for under this designation the summer in one of the large cities of the middle States is entitled to be spoken of, is well illustrated in the following memoranda of diseases of the digestive apparatus among children in Philadelphia and New York, for the years

1838 and 1839. In the former city, with a population of 200,000, the entire mortality of 1838 was 5118; of which the deaths of children from *cholera infantum* were 382: of these 364 were under two years, viz: 247 under a year, and 116 in the second year after birth. In 1839 the deaths from this disease were 230; the excess in 1838 being explained by the unusually long period of high atmospherical heat in the summer of that year. The number of children who thus perished within the first year from birth was 142; in the second 75; and between the termination of the second and the fifth year, 13. The entire mortality from the diseases of the digestive canal was as follows:

| | 1838. | 1839. |
|---|---|---|
| Cholera Infantum, - - - - - | 382 | 230 |
| Diarrhœa, - - - - - - - | 65 | 96 |
| Dysentery, - - - - - - | 45 | 68 |
| Inflammation of the Stomach and Bowels, | 80 | 83 |
| | 572 | 477 |

"In Washington, the proportionate mortality from cholera infantum is considerable, as might, *à priori*, be inferred from the excessive heat of its summers, without the mitigation of sea-breeze, by which even Charleston and Savannah are made more tolerable for infant life. Dr. Lindsly, in his article already referred to, gives us some useful information on this point, which is best conveyed in his own words.

" 'It will be seen by the following table, that of the whole number of deaths in the months of July and August, nearly one-half, and in two instance *more than one-half*, were under two years of age; and that of this number, almost three-fourths died of what is usually termed here 'summer complaint,' under which general term are included cholera infantum and simple diarrhœa of children. Also, that the cases were much more numerous in July and August, than in June, that a slight diminution took place in September, and that in October the number was again very small.

" 'The ratio of cases of infantile cholera in the above table is about the same as that exhibited by the record for several years past, and this may therefore be assumed as the proportion of victims annually destroyed by this fatal disease in Washington, during the months referred to.'

"In Boston, also, we still find that the summer gives a tropical climate, particularly for children under two years of age. For a period of ten years, from 1821 to 1830, inclusive, in which the entire number of deaths was 10,731, the mortality of children under two years, in the months of July, August, September, and October, was 1537; the whole mortality of this class, for the entire period, being 3182. Hence we see that the deaths of children between birth and two years old, in Boston, in the four months in which the summer temperature predominates, was just one-seventh of the entire number, and one-half of the particular class; or, in other words, the deaths are as numerous among children of this age in these four months, as in the remaining eight of the year. The deaths from cholera infantum, during the above period of ten years, was 149. Those from 1831 to 1839, or a period of nine yers, was 407; the annual average of twenty years was but a little over 27. This is quite a small proportion of deaths from cholera infantum, in a population averaging, during the entire period of twenty years, about 65,000, and which, in 1840, was 93,470. But if we add the kindred, and probably in most instances identical diseases, reported under the head of gastritis, teething, and dysentery, the amount of cases of deaths of children from gastro-intestinal disease, in the summer months in Boston, will be more easily accounted for; and place this city, on the score of infant mortality, in a line with, but quite behind, Philadelphia and New York. For much valuable information on the vital statistics of Boston, I refer you to the paper by Dr. Shuttuck in the *American Jour. Med. Sci.*, 1841.

"The aggravation of disease by the irritation of teething is manifest to every physician: it aggravates bronchitis in winter and cholera in summer: it might even be said to cause them, by inducing a morbid susceptibility—a predisposition to cold and moisture, in the former season, and to great heat in the second, without which these atmospherical extremes would be relatively innocuous. But that teething is only a secondary importance in the etiology of cholera, is manifest from the fact that, however suffering from this irritation at other seasons, rarely will children then have cholera."

We wish we could command room to give the entire lecture on Croup, by Dr. Bell, but we shall be obliged again to limit ourselves to a few extracts. Dr. Bell objects to the term *trachealis* as conveying a false idea of the pathology of the disease, and adduces very convincing evidence in favor of its being rather laryngeal than tracheal. His concluding arguments are that—

"We cannot understand, nor give any adequate explanation of the spasmodic croup, or of the fits of threatened suffocation in common croup, unless we admit the laryngeal seat of the disease. The irritating cause is in the mucous membrane of the glottis and larynx; by a reflex-motor action, the irritation of the membrane, transmitted to the brain, causes a return of innervation on the muscles of the glottis and larynx, and they are contracted with more or less violence. The cerebral excitement is kept up in these cases, and often augmented, 1. by the external air which is not in relation with the morbid sensibility of the inflamed organ; 2. by the duration of the inflammation itself; 3. by the various products of inflammatory secretion, which, like so many foreign bodies, irritate the air-passages. Hoarseness, or an equivalent condition of voice, may be the fixed one in croup; but the modifications depending on spasm of the laryngeal muscles must be considerable. The spasm may be continued, remittent, or intermittent; varieties which may exist in laryngitis with false membrane from the glottis down to the bronchia. A suspension of the more violent symptoms may take place, and the disease seem so far to be *intermittent;* but it is the spasm of the muscles, not the secretion of the exuded membrane and croup proper, which intermit. Inflammation of the membrane, like any other of the phlegmasiæ, is liable to exacerbation; but this latter does not always correspond with the spasm or fits of laborious breathing and imminent suffocation. The spasm of the glottis is most common at night; and hence a mucous irritant, hardly a source of complaint during the day, may, without any, or with very slight increment, be a source of imminent suffocation at night, owing to the greater susceptibility of the nerves and muscles at this time. Death rarely results from the occlusion of the glottis, by the thickening of the mucous membrane or the superposition of false membrane on this latter; but it may by the spasmodic action of the muscles. Even where the false membrane is formed and adherent, the breathing is sometimes free just before death.

"But, although the characteristic symptoms of croup depend on organic lesion of the larynx, we cannot render an account of all the phenomena of the disease if we overlook its tracheo-bronchial and even pulmonary complications. Of these I have already spoken. The dyspnœa gives a tolerable good measure of their presence and intensity. Hence, when we see the lips of a livid or violet color, the face tumefied, the eyes prominent and shining, headache, somnolency, comatose stupor, convulsions, a peculiar anxiety, hurried breathing, and throwing the head back, we recognize symptoms of impeded pulmonary circulation and decarbonization of the blood, and feel ourselves more urgently called upon to remove this state of things, whether the laryngeal symptoms proper be urgent or not."

The statistics of croup, given under the head of *mortality*, we venture to say, will surprise most of our readers, to whom this malady, formidable as it is, has not appeared by any means so intractable, as it would seem to be in many countries, and especially in France. Dr. Bell remarks:

"The mortality from croup has varied at different periods, and is rated very differently by writers in different countries. M. Andral estimates the recoveries to the deaths as barely one in ten; adding, that in a small village in France (near Treste-sous-Jouarre) during a period of epidemic croup, in 1825, of sixty children attacked, the entire number perished with the disease. It is encouraging to know that the treatment is more successful now, or, at any rate, the relative mortality is less than it was at the beginning of the present century. M. Double (*Traité du Croup*) has taken the pains to prepare a table exhibiting the results of the practice of fifty-eight writers who have published their experience in this disease, from which it appears that the number of cures is rather more than a third part of the whole. The authors being ranged in chronological order, we can make at once two classes, twenty-nine in each; and show that, whilst nearly four-fifths died of croup of those who had been attacked and who had been attended by the first class, not quite one-half of the entire number attended by the second, or more modern class, fell victims to the disease. In the spring of 1760, that of its first appearance in the county of Lancaster, England, Mr. Fell, a surgeon, who announces this fact, adds that, during the season, six children laboring under croup were committed to his care, to all of whom it proved fatal. But even at the present day our professional vanity is rebuked at the great mortality from croup in different parts of Europe. In a capital like Paris, where all the knowledge and resources of the art would, we might suppose, be brought to bear for the cure of disease in every form, the American student will be not a little surprised at the results of hospital practice, in croup, as exhibited in the prize essay on the subject by M. Boudet, (*Archiv. Gen. de Medicine*, *Fev. et Avril*, 1842.) Thus, of twenty-six cases of croup, received into the Children's Hospital in Paris, for a period of six years, or from 1834 to 1839, the deaths were twenty-two in number. In this year, (1839,) and in the two following years, croup was epidemic in Paris; the deaths from this disease having been in 1838, '39, and '40, respectively, 187, 286, and 326. In the Children's Hospital, in 1840, the deaths were twenty-three, and the recoveries but two; in 1840, during the first six months the deaths were twelve, being the entire number of all the cases received in the hospital. This terrible mortality might reasonably be attributed to the deteriorated constitution of the poor children brought to the hospital, did we not read in Boudet's essay that a physician, whom he names, (M. Loyseau,) living in Montmâtre, near to Paris, lost twelve out of fourteen cases, which he was called upon to attend at the houses of his patients. I have not the requisite data on which to express an opinion of the proportionate mortality of croup in the United States; but, adding my own experience to that of my professional friends, I should say, that it is not nearly so great as that given in any of the preceding statements of European authors, particularly the continental ones. Cases of croup are of very frequent occurrence with us—the deaths, compared to the number attacked, are few."

Is it not possible that this great mortality is ascribable, in part, at least, to the inefficiency of the plan of treatment adopted? Certain it is, that practitioners of our acquaintance, who have had large experience in croup, have cured a vastly larger proportion of their cases. We might mention, as among them, Dr. Cooke, lately of the Medical Institute of Louisville, who was in the habit of stating to his class, that he rarely lost a patient in that disease, and whose success was, probably, owing to the bold use of one of the remedies, calomel, which Dr. Bell so strongly commends. We give a portion of Dr. B.'s remarks on tartar emetic and calomel:

"You must by this time be fully aware of the therapeutical basis on which I rest my use of tartar emetic in croup, as well as in so many other of the phlegmasiæ. It is not merely as an emetic, but as a contrastimulant or sedative, opposed to inflammatory action irrespective of its procuring evacuations, that I habitually use this medicine. Its utility in this way is beginning to be perceived by some of the practitioners of Great Britain, one of whom, Dr. Wilson, of Kelso, relates his successful use of tartar emetic in croup; he having cured ten out of twelve cases. He gave, after leeches had been applied to the larynx, followed by warm poultices frequently renewed, the antimonial salt, in doses of one-fourth, to one-third of a grain, at first every hour, until a decided impression was made, and afterwards every two hours, till the patient was considered in safety. The toleration of the medicine did not extend so far as that it did not vomit at first quite freely; but it had no action on the bowels, which required castor oil or some other laxative to obviate costiveness. For children, Dr. Wilson properly directs from half a minim to a minim of laudanum in addition to the tartar emetic."

"It is not a little vexatious to find writers of established and deserved reputation take such limited views of the effects of *calomel* on various diseases. One will tell us that unless it purges it will do little good; another assures us that its administration will be useless in this stage of croup, because time is not allowed for it to touch the mouth. This last notion, that we cannot procure the full revolutionising and alterative effects of mercurial preparations in general, unless the salivary glands are inflamed and incipient ptyalism caused, is rank empiricism, and has completely blinded us to their therapeutical operation. Calomel, which we may speak of as in a great measure representing the other preparations of mercury, when taken into the stomach acts very speedily on the mucous membrane of this organ and of the small intestines; and, in a short time, on the liver and pancreas, which, by means of their excretory ducts, are placed in close and continuous relation with the intestinal mucous surface. Soon the large intestines are affected, and increased defecation is the consequence. But the operation of the medicine even in purgative doses is not confined to the gastro-intestinal canal and its subsidiary glands; it is extended to all the other mucous surfaces—the respiratory in one direction and the genito-urinary and its secretory apparatus in another; and is followed by increased expectoration and diuresis, together with an abatement of prior irritation which may have prevailed in one or other of these divisions. Calomel acts in a more especial manner on all portions of the mucous system, and through it on their glandular appendages; and hence its use is more immediately applicable to irritations and inflammations of the mucous membranes and their glands than to other forms of disease. Most mischievous has proved the notion that the general system is not affected by mercury, and notably by calomel, unless and until ptyalism is produced. Under the influence of this error, immense quantities of the medicine are introduced into the stomach, with the effect often of a great depression of the vital powers, and particularly of the functions of the nervous system, cold skin, excessive inertia, &c.; the prescribing physician all the while waiting for the action of the calomel. In this way the patient may be actually destroyed by mercury, without any suspicion being entertained of the fact by the doctors of the salivating school.

"Calomel, says Delafontaines, inspector-general of military hospitals at Warsaw, is the first and the most efficacious of all the remedies employed in croup. I regard it, he says, as a specific, at least as certain against croup as against syphilis. Albers and Olbers recommended and used calomel; sometimes alone, after venesection, sometimes alternating it with kermes mineral and musk. Frank, at Wilna, relies on calomel, after venesection, general and local. Autenrieth used it to act on the stomach and bowels as a revulsive, and to prevent the formation of a false membrane. Copious and fetid alvine discharges were followed in a surprising manner by a removal of the affections of the larynx. Dr. James Hamilton, the younger, gave a grain of calomel every hour to children within the year, and two grains and a half for those two years old, until relief was obtained; then he gradually diminished the dose. Commonly evacuations upwards and downwards resulted. A child, five months old, took thirty-two grains of calomel in twenty-four hours, and another took eighty-four grains in seventy-two hours. Two children were lost by the weakness which resulted from continuing the calomel after the symptoms of the croup had subsided. Drs. Kuhn, Redman, and Rush, gave calomel in large doses. Dr. Rush gave six grains two or three times a day. Dr. Physick gave thirty grains one day to the child three months old which was bled three times in the day. Bond first recommended it. Bayley used it. Bard also praised it, as augmenting the secretions and rendering them more fluid, and thus diminished or prevented the secretion and adhesion of the membrane. The use of calomel and enemata made up the chief treatment of Autenrieth in croup."

Pneumonia in infants is a most formidable disease, and at the same time is one in which the diagnosis is obscure. Dr. Bell very properly devotes particular attention to it in the following paragraph:

"*Symptoms of Infantile Pneumonia.*—The importance but yet difficulty of diagnosis of pulmonary inflammation in children and the frequency of the disease, will justify my making some additional remarks on this topic. Acceleration of the pulse and of respiration are important symptoms in the disease, and influence not a little our prognosis. When there is no complication of other acute diseases, they may be taken as a measure of the intensity and extent of the inflammation.

"At the outset of the disease there are some peculiarities in the mode of respiration, and, if the child be still fed from the breast, of sucking, mentioned by Dr. West, (*Memoir on Infantile Pneumonia—Brit. and For. Med. Rev.*,) which will aid us in forming a correct, and, what is of great importance, an early diagnosis. If, while a healthy infant is sleeping, the mouth be gently opened, it will be observed that the tongue is applied to the roof of the mouth, and that respiration is carried on through the nares. So soon, however, as the lungs become affected, even when no other symptom exists than general febrile disturbance, and perhaps the vomiting above alluded to, the infant will be seen no longer to breathe solely through his nose, but to lie with his mouth partly open, and drawing in air through it. This imparts to the tongue its preternatural dryness, and the same inability to respire comfortably through the nares causes the child to suck by starts. The infant seizes the breast eagerly, sucks for a moment with greediness, then suddenly drops the nipple, and, in many instances, begins to cry. As the disease advances, these peculiarities in the mode of sucking and respiration often become more striking, but it is at the onset of the disease that it is of especial importance to notice them, since they afford most valuable indications of its real nature.

"As respects the physical signs of infantile pneumonia, it may be said that the mucous rhonchus is heard in most cases in which catarrh has preceded the symptoms of pneumonia proper; but, Dr. West thinks, it should be looked on as one of the least important of the physical signs of this disease, since it was present in thirteen only of fifty-one children under five years of age. It is of importance, however, in the young subject, as the immediate precursor of bronchial respiration; while in the adult there is no such connection. 'The sub-crepitant rhonchus is a sign of far greater importance than the mucous rhonchus, whether we regard the frequency of its occurrence, or the consequences which follow it. It was heard in forty-two out of fifty-one cases; in thirty-one of which it either had not been preceded by mucous rhonchus, or if it had, that had ceased before the patients came (says Dr. West) under my notice.'

"The observation made by M. Guernard is confimed by Rilliet and Barthez, viz: that these sounds readily disappear if the little patients are kept seated for a short time; and that their greatest distinctness is when the child is raised from the bed."

This, we are aware, is a very imperfect notice of this elaborate and learned work, and we regret to be compelled to close it thus abruptly; but the arrangement of matter by our printer, leaves us no alternative. After all, perhaps, it is a matter of little moment, since these volumes will soon make their way into all the bookstores of the country, where readers will have an opportunity of judging of their merits for themselves. Our favorable opinion of them has already been expressed, and we will only add, that our estimate of their excellence has been heightened by repeated examinations. For all the qualities which the student or practitioner can desire in a work for study or reference, these lectures may be safely recommended, as comprising as much as could be brought within their limits of sound opinion and judicious practice. Y.

